# Associations between compulsive exercise and mental health constructs in eating disorders

**DOI:** 10.1186/s40337-025-01517-2

**Published:** 2026-01-03

**Authors:** Emelie Haglund, Nora Bouchta, Andreas Birgegård, Emma Forsén Mantilla, Cynthia M. Bulik, Emma Frans, Elin Monell

**Affiliations:** 1 Department of Clinical Neuroscience, Karolinska Institutet, Stockholm, Sweden; 2 Department of Medical Epidemiology and Biostatistics, Karolinska Institutet, Stockholm, Sweden; 3 Department of Physical Activity and Health, The Swedish School of Sport and Health Sciences, Stockholm, Sweden; 4 Department of Psychiatry, School of Medicine, University of North Carolina at Chapel Hill, Chapel Hill, USA; 5 Department of Nutrition, Gillings School of Global Public Health, University of North Carolina at Chapel Hill, Chapel Hill, USA

**Keywords:** Compulsive exercise, OCD, Cross-sectional study, Eating disorder, Perfectionism

## Abstract

**Background:**

Compulsive exercise (CE) is commonly observed in eating disorders (ED) and is associated with a more severe clinical picture. Including 3105 participants from the Eating Disorders Genetics Initiative-Sweden, the aim of this cross-sectional study was to expand the knowledge of how CE relates to ED symptoms and other core psychological features.

**Methods:**

Through multiple linear regression analyses, we investigated simple and unique associations between CE, measured with the Compulsive Exercise Test (CET) including its subscales and ED symptoms, obsessive compulsive disorder (OCD) symptoms, anxiety, perfectionism, depression, and health-related quality of life.

**Results:**

Results suggested that ED symptoms, OCD symptoms, and perfectionism all have unique positive associations with CE, and depressive symptoms a negative association when controlling for the other constructs. Each CET subscale showed its own specific pattern of associations with the examined constructs.

**Conclusions:**

Overall, results were consistent with previous research and the proposed cognitive-behavioral model of CE. Implications support the idea that CE may not be clinically relevant in the absence of ED symptoms, but an important symptom domain when they are present. Future research should focus on the directionality of associations between OCD symptoms, perfectionism, and CE, including samples without ED experience.

**Supplementary Information:**

The online version contains supplementary material available at 10.1186/s40337-025-01517-2.

## Abstract (plain language summary)

Compulsive exercise is a common symptom in eating disorders, but less is known about its associations with other core psychological features including obsessive compulsive disorder (OCD) symptoms, anxiety, perfectionism, depression, and health-related quality of life. Even less is known about associations between specific aspects of compulsive exercise and these other features. Such knowledge could be important in treatment. In this study, we examined these associations in 3105 individuals with ongoing eating disorder pathology. We found that higher levels of compulsive exercise were uniquely associated with more eating disorder symptoms, perfectionism, and OCD symptoms. We also found some unique associations between specific compulsive exercise aspects and other core psychological features.

**Supplementary Information:**

The online version contains supplementary material available at 10.1186/s40337-025-01517-2.

## Background

Compulsive exercise (CE) is present in around 50% of individuals with eating disorders (ED) [1–3], and is associated with ED pathology [1, 4, 5] and ED symptom severity [2, 3]. Further, CE is associated with suicidal behavior, psychiatric comorbidity, and worse treatment outcomes in patients with ED [2, 3, 6, 7]. CE can be defined as exercise that has compulsive features, in terms of it being performed to relieve distress associated with perceived negative consequences of not exercising and is pursued to the detriment of other valued life contents and despite illness or injury [8, 9]. CE is an aggravating symptom and learning more about its components and correlates is necessary to further develop existing treatment interventions (e.g., [10]). In this study, we investigated associations between core constructs of CE and adjacent well-established and validly measured psychiatric symptoms including obsessive-compulsive disorder (OCD) symptoms, clinical perfectionism, anxiety, depression, and health-related quality of life (HRQoL) to shed further light on the clinical importance of CE.

There are several conceptualizations of problematic exercise, but research suggest prioritizing CE since it captures significant variance in ED symptoms [5]. In a cognitive-behavioral model of CE in ED, Meyer and colleagues [11, 12] conceptualize CE as maintained by eating pathology, emotion dysregulation, compulsivity, perfectionism, and behavioral rigidity. According to this empirically based model, CE in the context of ED may function to reduce anxiety tied to food intake and/or to impact body shape and weight. CE is thus negatively reinforced by allowing the individual to avoid or reduce negative emotions (i.e., anxiety, guilt, and shame) tied to ED cognitions and behaviors [8, 9]. Further, traits such as rigidity and perfectionism are proposed to negatively influence intensity and frequency of exercise patterns, development and adherence to CE rules, and the course and maintenance of CE. In this model it is also proposed that CE in the absence of ED symptoms lacks clinical relevance [8].

The central psychological constructs maintaining CE are all thought to be captured by the Compulsive Exercise Test (CET; [12] and its subscales. It consists of five subscales: avoidance and rule-driven behavior (henceforth Avoidance/Rules), weight control exercise (Weight Control), mood improvement (Mood), lack of exercise enjoyment (Lack of Enjoyment), and exercise rigidity (Exercise Rigidity). The Avoidance/Rules subscale measures avoidance of perceived negative consequences of not exercising, Weight Control measures the extent to which exercise is used to control body weight, Mood measures exercise as mood improvement or stress/anxiety management, Lack of Enjoyment measures lack of enjoyment or interest in physical activity, and Exercise Rigidity measures inflexibility regarding exercise.

CE and excessive exercise, a term signifying problematic exercise but not measured using dedicated CE measures, has been associated with OCD symptoms in individuals with current ED [7, 13, 14] and with higher perfectionism, in both non-clinical [15, 16] and clinical samples [2, 13]. No study has yet examined the association between perfectionism or OCD symptoms and the CET and its subscales in individuals with a range of ED.

Associations between CE and anxiety and depression are less clear. Excessive exercise has been associated with higher trait anxiety in broad ED [13] and with both higher trait anxiety and depression in adolescents with anorexia nervosa (AN) [17]. Maladaptive exercise (exercise that leads to negative consequences and/or disrupts daily functioning) has been associated with higher prevalence of both lifetime and current anxiety and depression in individuals with both current and lifetime ED [7]. However, in a large Swedish study [2] weight control exercise was shown to be significantly associated with anxiety among ED patients, but with a negligible effect size. CE appears to be associated with depression in fitness instructors [18] and in professional and recreational exercisers [19].

High CET scores have been associated with psychological distress (including both anxiety and depressive symptoms) among patients with AN [20]. However, in another study on CET and anxiety in adolescents with AN, no association was observed [21]. Further, CET scores were negatively associated with depressive symptoms among university students [22], while among recreational exercisers and athletes, CET subscales Avoidance/Rules and Lack of Enjoyment were positively associated with depression whereas the Exercise Rigidity subscale showed a negative association [23]. No study has yet investigated the CET (including subscales) in relation to generalized anxiety or depression in ED samples.

Regarding quality of life, The Eating Disorder Quality of Life (EDQoL) seems consistently negatively associated with CE in AN [20, 24]. Associations between the CET subscales (the maintaining factors of CE) and EDQoL have not yet been explored in broad ED samples.

In sum, CE and different exercise related constructs have shown some associations with OCD symptoms, perfectionism, anxiety, depression and QoL, although findings have been inconsistent. Further, only some concepts have been examined in broader ED samples, and only a few studies have used the CET in such research.

### Aims

We aimed to examine, in a large ED sample, associations between the CET including subscales and ED symptoms, OCD symptoms, anxiety, perfectionism, depression, and HRQoL to clarify relationships between the constructs and add specificity regarding associates of the factors that maintain CE. This knowledge may inform future research exploring causal relationships and carry implications for intervention targets. This study is the first to investigate the unique contributions of multiple psychological measures to CET variance in individuals with current ED. Based on previous findings, hypotheses were that there would be positive associations between the CET and ED symptoms, OCD symptoms, and perfectionism. Due to inconsistent findings in the literature, no hypotheses were formulated regarding generalized anxiety, depressive symptoms, and HRQoL. Lastly, no hypotheses were formulated regarding the associations between the CET subscales and the included measures due to lack of previous studies.

## Methods

### Recruitment and procedure

The present study used data from the Eating Disorders Genetics Initiative-Sweden (EDGI-SE) study (methods paralleled the international EDGI study described elsewhere [25]). Data collection was conducted from February 2021 to May 2023. Recruitment of participants with current or past ED was conducted through recontact of previous study participants [26, 27], via Swedish National quality registers for ED (Riksät/Stepwise; [28]) and by advertisements at ED clinics, sports facilities, websites, and social media. All recruitment channels led participants to www.edgi.se, where participants consented using a Swedish digital identification method (BankID) and completed online questionnaires. A range of data was collected and not all will be considered here, including genetic information [25]. Participants were awarded with two cinema tickets after participation. The study was approved by the Swedish Ethical Review Authority (DNR: 2020–02243 and 2023-00254-02).

Inclusion required: [1] Have a current or past ED [2], be at least sixteen years old [3], live in Sweden and understand Swedish well enough to answer the questionnaires, the study material, and provide informed consent.

### Participants

**Fig. 1:**
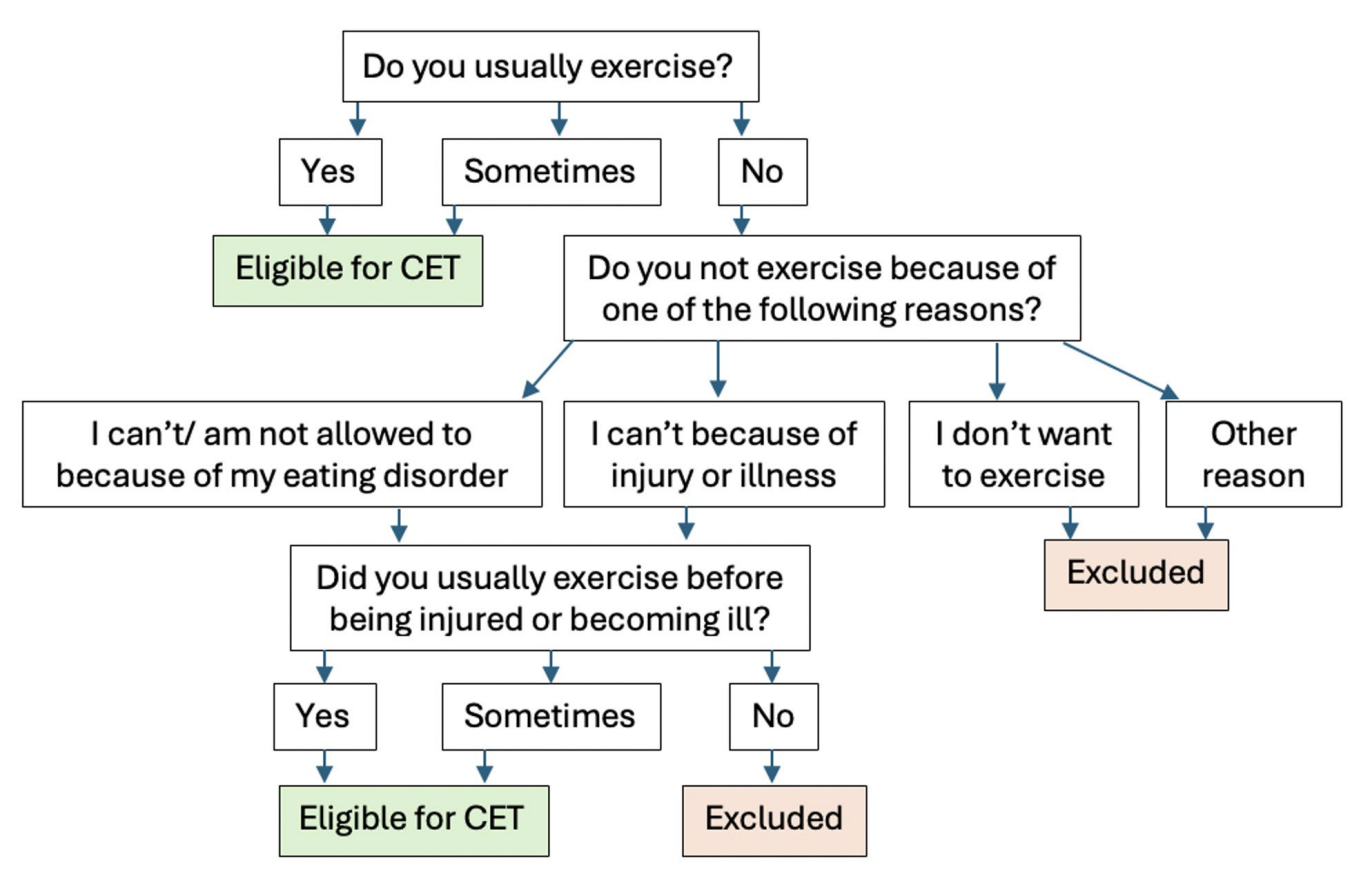
Flowchart for ED100K.v3 items determining eligibility to fill out the CET. “Excluded” refers to exclusion from the present study only

Of 8,796 individuals with current or past ED according to the ED100K.v3 (see below), 5,260 did not score in the clinical range on the Eating Disorders Examination Questionnaire (EDE-Q; see below), 429 were not invited to complete the CET (survey decision logic in Fig. 1), and two were 15 years old (minimum age for participation was 16), yielding a final sample of 3,105 individuals, of which the vast majority (97.8%) were female. A majority of the sample (74.6%) answered a question on monthly income. Of those respondents, 2.8% reported that they did not know, 23.9% reported earning less than 10,000 SEK, 45.1% reported earning 10,000–29,999 SEK, 24.8% reported earning 30,000–49,999 SEK, and 4.23% reported earning more than 50,000 SEK (1 EUR ≈ 11.8 SEK, as of September 12, 2025). Mean EDE-Q global score was 4.0 (*SD* = 0.78) which indicates ongoing ED concerns. Most met ED100K.v3 criteria for lifetime AN (62%), followed by bulimia nervosa (BN; 17%), other specified feeding and ED (OSFED; 17%), and binge eating disorder (BED; 4%). The diagnostic grouping was conducted hierarchically, where individuals who at any point met the criteria for AN, were assigned to the AN group, followed by BN, BED, and OSFED. All lived in Sweden at the time data was collected and spoke either Swedish or English. Mean age was 33.1 years (*SD* = 11.55; range 16–79) and mean BMI = 24.4 kg/m^2^ (*SD* = 6.54; range 12.1–59.9).

### Measures

All measures are self-reported and included in the online EDGI survey. Internal consistencies in the present sample are presented in Table 1.

*CET* (introduced above) contains 24 items answered on a 6-point Likert scale (0–5) where a higher score suggests more severe CE [12]. The clinical cutoff for individuals with current ED has been suggested at ≥ 15 points, with acceptable sensitivity (0.78), specificity (0.73), and positive predictive value (0.75) [29]. The CET total scale and subscales have previously shown acceptable to excellent internal consistency in ED, except for the Lack of Enjoyment scale where Cronbach’s alpha (α) was 0.62 [29].

*ED100K.v3* is a self-report measure designed to screen for lifetime DSM-5 ED modeled after the Structured Clinical Interview for DSM-5 [30]; since EDGI aimed to study genetic (i.e. lifelong) factors behind ED, lifetime diagnoses were targeted. The scale contains 84 items, with acceptable validity [25, 26].

*The Eating Disorders Examination Questionnaire* version 6.0 (EDE-Q) addresses ED symptoms during the past 28 days [31]. It includes 28 items, 6 of which gauge frequency of ED behaviors and the remaining 22, scored 0–6, can be averaged to yield a global score of cognitive symptoms. Higher scores indicate more severe ED pathology, and good internal consistency in ED has been reported (α = .90) [32]. In the present study, a cut-off of 2.8 to distinguish a clinical from a non-clinical range was used [33, 34]. Present internal consistency was calculated using the entire sample, not only the clinical range in focus in this study.            

*The Revised Obsessive-Compulsive Inventory* (OCI-R) measures OCD symptoms over the past month using 18 items scored 0–4 (higher = more symptoms). Summing the item scores provides a total score. The OCI-R has previously shown good internal consistency (α > 0.81) [35, 36].

*Generalized Anxiety Disorder 7* item scale (GAD-7) screens for generalized anxiety disorder (GAD) symptoms during the last two weeks in 7 items rated 0–3 (higher = more symptoms) with excellent internal consistency [37].

*The Multidimensional Perfectionism Scale* (MPS) consists of 35 items and six subscales: concern over mistakes, personal standards, doubts about actions, parental criticism, parental expectations, and organization [38]. Higher scores on each subscale indicates more perfectionism. In the present study, 12 of the 35 items from three of the six subscales were used; the 4 items with the highest factor loadings from the doubts about actions, personal standards, and concern over mistakes subscales, respectively [39]. Internal consistency of this revised version of the MPS has not been reported previously.

*The Patient Health Questionnaire* (PHQ-9) assesses depressive symptoms using 9 items that correspond to DSM-criteria for major depressive disorder, rated 0–3 and yielding a sum score where higher scores indicate more depressive symptoms. High internal consistency has previously been reported [40, 41].

*EDQoL* has 25 items scored 0–4 with 4 subscales and an average total score of health-related quality of life in ED, where higher scores indicate poorer quality of life. High internal consistency has been found for EDQoL [42].

*Body Mass Index* (BMI), *Sex*, and *Age* were used as covariates; BMI was calculated based on self-reported current weight and height, and sex was biological sex assigned at birth (female coded 0 and male 1).

### Statistical analyses

Jamovi (version 2.3) was used for all analyses [43]. Skewness and kurtosis for all variables were assessed for normality and no problems were found based on recommendations [44]. To assess multicollinearity, Variance Inflation Factor (VIF) scores were examined, with the highest VIF < 2.35. Regression analyses with CET total score as dependent variable were conducted in four steps: (1) only covariates entered, (2) eight multiple linear regression analyses where one of the eight measures (EDE-Q, OCI-R, GAD-7, MPS, PHQ-9, and EDQoL) was added to covariates, (3) variables significantly associated with CET in Step 2 were entered together to examine unique associations with the CET, and (4) variables with unique associations to the CET from Step 3 were further examined in five multiple regression analyses for their associations to CET subscales. Analyses were adjusted for age, sex, and BMI. Although previous research has found little correlation between age and CE [29, 45], minor effects have been found such that individuals who engage in CE are slightly younger [2, 13], explainable by exercise being the most accessible way to compensate for energy intake among young individuals [13]. CE appears to occur at approximately equal rates among both men and women with ED [2], and it also seems associated with lower BMI in individuals with ED [2, 13]. Correlations and beta (β) coefficients were interpreted as large ≥ 0.50, moderate ≥ 0.30, and small ≥ 0.10. Correction for multiple testing was undertaken using the Bonferroni-Holm procedure.

## Results

Descriptive data including Cronbach’s alpha are shown in Table 1, where all measures showed at least acceptable internal consistency. Correlations between independent variables and covariates are shown in Table S1. Differences in independent variable scores between the AN diagnostic group and the others combined is presented in Table S2.

**Table 1:** Cronbach’s alpha and descriptive statistics for independent and dependent variables

Measure	Cronbach’s α	M (SD)	Range (min–max)
CET total	0.88	14.5 (3.33)	4.1–24.4
Avoidance/Rules	0.93	2.8 (1.31)	0.0–5.0
Weight Control	0.78	3.6 (1.00)	0.0–5.0
Mood	0.89	3.7 (1.11)	0.0–5.0
Lack of Enjoyment	0.88	1.9 (1.34)	0.0–5.0
Exercise Rigidity	0.72	2.5 (1.33)	0.0–5.0
EDE-Q	0.96^†^	4.0 (0.78)	2.8–6.0
OCI-R	0.90	21.5 (14.00)	0.0–72.0
GAD-7	0.90	10.1 (5.75)	0.0–21.0
MPS	0.87	41.3 (9.51)	12.0–60.0
PHQ-9	0.87	14.1 (6.43)	0.0–27.0
EDQoL	0.92	1.7 (0.65)	0.0–4.0

*CET* Compulsive Exercise Test,* EDE-Q* Eating Disorder Examination Questionnaire,* OCI-R* Obsessive Compulsive Inventory Revised,* GAD-7* 7 item Generalized Anxiety Disorder scale,* MPS*  Multidimensional Perfectionism Scale, *PHQ-9 * 9 item Patient Health Questionnaire,* EDQoL* ED Quality of Life. ^†^For the EDE-Q, internal consistency was calculated without excluding participants with scores below 2.8 to avoid issues with restricted range

### Regression analyses

In Step 1, BMI was negatively associated with the CET with a small effect size (*p* < .001; *β*=-0.21), while the other covariates were not statistically significant (Table S3). In Step 2 (Table 2, where covariate results are not shown; full results in Table S4), all independent variables were significantly associated with the CET even after inclusion of covariates. The models including the EDE-Q and MPS explained 17% and 13% of the total variance, respectively, with moderate effects for each independent variable, while the remaining models explained 7–10% of the total variance with small effects for those independent variables. More CE indicated more ED severity, OCD symptoms, generalized anxiety, perfectionism, depressive symptoms, and worse EDQoL.

**Table 2:** Second step of analyses. Six multiple regression analyses with CET as dependent and each independent variable, including covariates in every model

	b	SE	$\:{\upbeta\:}$	t	*p*	*R*	*R* ^2^	Adj. *R*^2^	ΔR^2^
EDE-Q	1.50	0.07	0.35	21.46	< 0.001*	0.41	0.17	0.17	0.124
OCI-R	0.06	0.00	0.24	13.55	< 0.001*	0.31	0.10	0.10	0.054
GAD-7	0.12	0.01	0.20	11.51	< 0.001*	0.29	0.08	0.08	0.039
MPS	0.11	0.01	0.31	17.73	< 0.001*	0.36	0.13	0.13	0.088
PHQ-9	0.09	0.01	0.17	9.54	< 0.001*	0.27	0.07	0.07	0.027
EDQoL	1.19	0.09	0.23	13.46	< 0.001*	0.31	0.10	0.09	0.053

In step 3 (Table 3), the model explained 22% of the total variance where the EDE-Q (moderate effect size), OCI-R (less than small effect), MPS (small effect), and PHQ-9 (small effect) were significantly associated with the CET. Thus, when all other factors were held constant, more CE significantly indicated more ED severity, OCD symptoms, perfectionism and less depressive symptoms. For Step 3, a complementary analysis was also conducted without males which did not change the results in any significant way (see Table S5).

**Table 3:** Third step of analyses. Multiple regression analysis with CET Total as dependent variable

	b	SE	$\:\beta\:$	t	*p*	sr^2^	*R*	*R* ^2^	Adj. *R*^2^
CET							0.47	0.22	0.22
EDE-Q	1.26	0.08	0.30	14.91	< 0.001*	0.056			
OCI-R	0.02	0.00	0.09	4.53	< 0.001*	0.005			
GAD-7	0.03	0.01	0.06	2.45	0.014	0.002			
MPS	0.07	0.01	0.20	10.45	< 0.001*	0.028			
PHQ-9	– 0.06	0.01	− 0.12	– 4.87	< 0.001*	0.006			
EDQoL	0.08	0.12	0.02	0.69	0.489	< 0.001			
BMI	– 0.10	0.01	− 0.19	– 11.42	< 0.001*	0.033			
Age	0.02	0.01	0.06	3.64	< 0.001*	0.003			
Sex	0.35	0.36	0.02	0.97	0.331	< 0.001			

In Step 4 (Table 4) concerning unique associations between independent variables and CET subscales (again adjusting for covariates), the EDE-Q, OCI-R, and MPS were positively associated with the Avoidance/Rules subscale. The model explained 21% of the total variance and effects were small for EDE-Q and MPS and less than small for OCI-R. For Weight Control, the model explained 20% of the total variance and the EDE-Q and MPS showed moderate and small positive associations, respectively, and PHQ-9 showed a negative, less than small effect. For the Mood subscale, the model explained 7% of the total variance and MPS showed a positive and less than small association, and PHQ-9 negative and small. The PHQ-9 was however positively associated with the Lack of Enjoyment subscale, with a small effect where the model explained 10% of the total variance. Finally, for the Exercise Rigidity, the model explained 13% of the total variance and EDE-Q, OCI-R, and MPS showed positive associations (small effects) and PHQ-9 a negative association, also with a small effect. In each model, BMI also showed a negative and small or less than small association, except for the Lack of Enjoyment subscale where it was positive and small. Age was negatively associated with Weight Control and positively with Mood (both less than small effects).

**Table 4:** Fourth step of analyses. Multiple regression analyses with CET subscales as dependent variables and the uniquely associated variables and covariates as independent variables

	b	SE	$\:\beta\:$	t	*p*	sr^2^	*R*	*R* ^2^	Adj. *R*^2^
Avoidance/Rules							0.46	0.21	0.21
EDE-Q	0.42	0.03	0.25	13.84	< 0.001*	0.049			
OCI-R	0.01	0.00	0.09	4.41	< 0.001*	0.005			
MPS	0.02	0.00	0.17	8.86	< 0.001*	0.020			
PHQ-9	– 0.01	0.00	− 0.04	– 2.17	0.030	0.001			
BMI	– 0.06	0.00	− 0.28	– 17.05	< 0.001*	0.074			
Age	0.01	0.00	0.05	2.95	0.003	0.002			
Sex	0.14	0.14	0.02	0.98	0.326	< 0.001			
Weight Control							0.45	0.20	0.20
EDE-Q	0.48	0.02	0.38	20.76	< 0.001*	0.112			
OCI-R	0.00	0.00	0.04	2.22	0.027	0.001			
MPS	0.01	0.00	0.14	7.23	< 0.001*	0.014			
PHQ-9	– 0.01	0.00	− 0.08	– 4.20	< 0.001*	0.005			
BMI	– 0.01	0.00	− 0.09	– 5.09	< 0.001*	0.007			
Age	– 0.01	0.00	− 0.08	– 4.65	< 0.001*	0.006			
Sex	0.03	0.11	0.00	0.29	0.769	< 0.001			
Mood							0.27	0.07	0.07
EDE-Q	0.04	0.03	0.03	1.44	0.150	< 0.001			
OCI-R	– 0.00	0.00	− 0.03	– 1.42	0.156	< 0.001			
MPS	0.01	0.00	0.07	3.55	< 0.001*	0.004			
PHQ-9	– 0.04	0.00	− 0.20	– 9.68	< 0.001*	0.028			
BMI	– 0.03	0.00	− 0.18	– 10.02	< 0.001*	0.030			
Age	0.01	0.00	0.10	5.46	< 0.001*	0.009			
Sex	0.05	0.13	0.01	0.42	0.677	< 0.001			
Lack of Enjoyment							0.32	0.10	0.10
EDE-Q	0.04	0.03	0.02	1.30	0.194	< 0.001			
OCI-R	0.00	0.00	0.04	1.71	0.087	< 0.001			
MPS	0.00	0.00	0.03	1.61	0.108	< 0.001			
PHQ-9	0.04	0.00	0.19	9.26	< 0.001*	0.025			
BMI	0.04	0.00	0.22	12.21	< 0.001*	0.043			
Age	0.00	0.00	0.03	1.75	0.080	< 0.001			
Sex	– 0.08	0.16	− 0.01	– 0.54	0.591	< 0.001			
Exercise Rigidity							0.36	0.13	0.13
EDE-Q	0.31	0.03	0.18	9.69	< 0.001*	0.026			
OCI-R	0.01	0.00	0.13	6.35	< 0.001*	0.011			
MPS	0.02	0.00	0.15	7.39	< 0.001*	0.015			
PHQ-9	– 0.03	0.00	− 0.13	– 6.18	< 0.001*	0.011			
BMI	– 0.04	0.00	− 0.21	– 11.84	< 0.001*	0.039			
Age	0.00	0.00	0.04	2.04	0.042	0.001			
Sex	0.20	0.15	0.02	1.30	0.194	< 0.001			

## Discussion

The present study examined, in a predominately female sample, both the simple and unique associations between CE and six psychiatric/psychological factors and explored their respective associations with the five proposed maintaining factors of CE. Based on previous research it was hypothesized that CE would be associated with ED symptoms, OCD symptoms, and perfectionism.

Indeed, CE was positively and uniquely associated with ED symptoms, in line with previous research [2, 3, 6]; this pertained particularly to Avoidance/Rules, Weight Control, and Exercise Rigidity. Previous research has shown Weight Control to be associated with ED severity [4, 24], and we found this also for Avoidance/Rules and Exercise Rigidity, possibly due to our larger sample size. These findings partially align with the theoretical framework of CE [11], where weight and shape concerns in ED pathology are central drivers of CE, although they also reciprocally reinforce each other. The model further suggests that avoidance of adverse withdrawal effects (i.e., anticipated negative consequences such as anxiety) and rigidity separately maintain CE and reinforce ED pathology, thus additionally contributing to CE indirectly. Our results are consistent with these ideas since Weight Control was moderately associated with ED severity, as were Exercise Rigidity and Avoidance/Rules with small effect sizes. We did not find this for Lack of Enjoyment or Mood, which would have been expected based on the theory [11].

Further, we found statistically significant associations between OCD symptoms measured with the OCI-R and the total CET score, in line with previous findings [2, 13], and specifically with the Avoidance/Rules and Exercise Rigidity subscales. This is inconsistent with a previous study [17] where no association between obsessive-compulsiveness and excessive exercise was found in a small AN sample. One explanation that the authors discuss is that obsessive-compulsiveness is associated with exercise cognitions rather than level of excessive exercise, and the CET is more targeted toward thoughts around exercise than the measure of physical activity used in that study.

Regarding perfectionism, we found positive associations between the CET and MPS, in line with previous findings [2, 4, 13, 16], and specifically implicated were Avoidance/Rules, Weight Control, Mood, and Exercise Rigidity. Weight Control was previously found to most strongly explain variance in the MPS worry about mistakes subscale in non-clinical samples [4, 16]. However, when using an instrument measuring clinical perfectionism instead of MPS, Egan et al. [16] found associations also with the Avoidance/Rules, Weight Control, and Mood subscales, suggesting a more general association between perfectionism and CE and that perfectionism may contribute to CE regardless of the presence of ED. This may indicate that reducing perfectionism is relevant for reducing CE in both individuals with and without ED.

The positive association between the CET and GAD-7 observed in Step 2 was attenuated when simultaneously entering all significant variables in Step 3, suggesting that other variables better accounted for this variance. Based on the correlation patterns, ED severity, OCD symptoms, perfectionism, and depressive symptoms may be implicated. Generalized anxiety may still be a maintaining factor in CE, but it is possible that anxiety could stem from other psychological drivers. Previous studies [2, 13] found that excessive exercise was associated with anxiety, but without controlling for other constructs. Holtkamp et al. [17] found similar results and did control for BMI, food restriction, body image/slimness ideal, depression and obsessive-compulsiveness, but not for perfectionism, which we found to be moderately correlated with generalized anxiety. CE may be a coping-strategy to manage the anxiety in ED [11, 17], and our results add that CE may potentially regulate anxiety stemming from obsessive-compulsiveness or perfectionism. Regarding depressive symptoms, the positive association between CET and PHQ-9 in Step 2 became negative in Step 3, specifically with Weight Control, Mood and Exercise Rigidity in Step 4. Speculatively, CE may function as maladaptive emotion regulation, thus attenuating depressive symptoms, in the presence of ED symptoms. Our Step 2 results are thus reminiscent of the Liao et al. [7] finding that CE was associated with higher depression scores, but when controlling for the other outcomes, this was reversed. Also, that study used a different sample (individuals with both lifetime and current ED were included). There was however still a positive association between depressive symptoms and Lack of Enjoyment; to the extent that the exercise is a burden rather than enjoyable, the emotion regulating function may be attenuated. Further, Cosh et al. [23] also found a positive association between depressive symptoms and the Lack of Enjoyment subscale, speculating that Lack of Enjoyment items correspond to depressive symptoms (e.g. “I find exercise a chore”; “I do not enjoy exercising”).

EDQoL results were reminiscent of GAD-7, with positive associations with the CET in Step 2 disappearing in Step 3. Focusing mainly on the EDQoL, results again indicate that other variables captured the variance, and based on correlations, ED symptoms, OCD symptoms, perfectionism, and/or depressive symptoms may be relevant here. Mond et al. [46] also found associations between exercise variables and QoL that were not significant when ED severity was statistically accounted for. Young et al. [20] and Harris et al. [24] also found positive associations between CE and QoL but did not statistically account for variance in ED severity. Thus, findings suggest that concurrent psychopathology, rather than CE, is associated with worse QoL.

### Implications

Our results are broadly consistent with the cognitive-behavioral model of CE [11] by particularly supporting the association between CE and ED symptoms, and also associations with perfectionism and obsessive-compulsiveness. Further, our results suggest that while related to CE, depressive symptoms, generalized anxiety, and QoL may be better explained by variations in ED, OCD symptoms, and/or perfectionism. When the other factors were held constant, more CE even indicated lower depression scores, consistent with previous findings in university students [22]. Theoretically, this would support Meyer et al. [11], who state that CE without ED has no clinical relevance. This may also imply that CE is less useful as a stand-alone diagnosis, at least according to the definition by Meyer et al. [8, 10] and/or measured by the CET.

Our findings further underscore the importance of assessing and systematically treating CE in EDs since it is associated with both ED symptoms and other problematic characteristics, possibly acting as a maintaining factor, or at least as a signal of clinical severity. Previous findings also implicate that patients who either continue to engage in problematic exercise or start exercising during the first year of treatment achieve remission only half as often as those who stop or have never exercised [2, 3]. Traits of obsessive-compulsiveness and perfectionism may be played out in CE as for instance, rigid exercise routines, a sense of never being active enough and an intense fear of consequences related to not being active or changing one’s routine. Such cognitions and behaviors related to obsessive-compulsiveness and perfectionism in CE, coupled with a focus on how ED symptoms and CE relate, most likely need to be recognized and targeted in treatment for CE to cease.

### Strengths and limitations

Strengths include the large sample, nationwide recruitment, valid and reliable measurement methods, and breadth of constructs assessed, allowing us to investigate unique contributing factors statistically. Limitations include the cross-sectional nature of the study precluding causal interpretations or longitudinal changes in the constructs. Our study may help motivate the use of measures on perfectionism, ED symptoms, depression and OCD symptoms in CE treatment research. Although, without information on directionality, it cannot provide clinically useful information, such as points on treatment focus. Further limitations include limited generalizability due to the small proportion of males and the overrepresentation of individuals who had AN at some point in their lives, neither of which mirrors epidemiological expectations [47]. The latter may have been partly countered by adjusting analyses for BMI, assuming that a history of AN suggests present AN, but this is an incomplete control for this imbalance. Relatedly, although the EDGI-SE used a wide array of recruitment strategies, all participants actively chose to take part in the study which may introduce some unknown bias potentially threatening the generalizability. Further, the study relied on self-report measures, with well-known possible biases including lack of illness insight, which could have impacted our sample as we set the EDE-Q cut-off at 2.8 to determine clinical status. Thus, we may have excluded some individuals who were currently more symptomatic than their EDE-Q scores suggested. As shown in previous studies of problematic exercise in ED however [2, 3], scores on other self-report measures are likely to have been similarly attenuated, increasing the risk that results would not have been valid had they been included.

## Conclusions

Our findings suggest that higher CE scores are uniquely associated with more ED symptoms, perfectionism, and OCD symptoms, especially in females with a history of AN given the composition of our sample. Results confirm the important role of CE in ED and overall support the cognitive-behavioral CE model [11], including the interpretation that CE may not be clinically relevant without the presence of ED symptoms. Nevertheless, future research could further investigate if perfectionism and obsessive-compulsiveness could “suffice” to raise CE or some other form of problematic exercise to a clinically relevant stand-alone condition, since our lack of a subsample without ED experience precluded evaluation of this issue. Further, future research should prioritize longitudinal studies to disentangle causality of the associations studied here as well as evaluate associations in different diagnostic groups.

## Supplementary Information

Below is the link to the electronic supplementary material.

Supplementary Material 1.

## Data Availability

According to Swedish regulations, deidentified data used in this study are available from the authors.

## Abbreviations

AN: anorexia nervosa
BN: bulimia nervosa
BED: binge eating disorder
BMI: Body Mass Index
CE: compulsive exercise
CET: Compulsive Exercise Test
ED: eating disorder
EDE-Q: Eating Disorders Examination Questionnaire version 6.0
EDGI-SE: Eating Disorders Genetics Initiative-Sweden
GAD-7: Generalized Anxiety Disorder 7 item scale
HRQoL: health-related quality of life
MPS: Multidimensional Perfectionism Scale
OCI-R: Revised Obsessive-Compulsive Inventory
OSFED: other specified feeding and ED
PHQ-9: The Patient Health Questionnaire
VIF: Variance Inflation Factor
