# Supplementary material for: Associations between compulsive exercise and mental health constructs in eating disorders
Source: J Eat Disord. 2026 Jan 3;14:32. doi: 10.1186/s40337-025-01517-2 (PMC12849285; doi:10.1186/s40337-025-01517-2)
Supplement: Supplementary file 1 — Supplementary Material 1. [file 40337_2025_1517_MOESM1_ESM.docx]

**Supplementary tables**

Table S1. *Pearson Correlation Matrix between Independent Variables and Covariates*

| Variable | 1 | 2 | 3 | 4 | 5 | 6 | 7 | 8 | 9 |
| --- | --- | --- | --- | --- | --- | --- | --- | --- | --- |
| 1. BMI | **—** |  |  |  |  |  |  |  |  |
| 2. Age | **.25*** | — |  |  |  |  |  |  |  |
| 3. Sex | **.11*** | .08* | — |  |  |  |  |  |  |
| 4. EDE-Q | .00 | .01 | -.00 | — |  |  |  |  |  |
| 5. OCI-R | -.06* | **-.25*** | .04 | **.26*** | **—** |  |  |  |  |
| 6. GAD-7 | **-.11*** | **-.25*** | -.01 | **.32*** | **.51*** | **—** |  |  |  |
| 7. MPS | **-.12*** | **-.26*** | .00 | **.28*** | **.47*** | **.42*** | — |  |  |
| 8. PHQ-9 | -.01 | **-.13*** | -.00 | **.43*** | **.45*** | **.67*** | **.39*** | — |  |
| 9. EDQoL | -.02 | -.09* | .00 | **.57*** | **.36*** | **.46*** | **.32*** | **.60*** | **––** |

*Significant after Bonferroni-Holm correction. BMI = Body Mass Index; EDE-Q = Eating Disorder Examination Questionnaire; OCI-R = Obsessive Compulsive Inventory Revised; GAD-7= 7 item Generalized Anxiety Disorder scale; MPS = Multidimensional Perfectionism Scale; PHQ-9 = 9 item Patient Health Questionnaire; EDQoL = ED Quality of Life.

Table S2. *One-Way ANOVA, Evaluating Differences in CET, EDE-Q, OCI-R, Between Hierarchical Lifetime Diagnosis of AN with the Other Diagnostic Groups Combined (Bulimia Nervosa, Binge Eating Disorder and Other Specified Feeding and Eating Disorders)*

|  | *F* | *df1* | *df2* | *p* | *N* | *M* | *SD* |
| --- | --- | --- | --- | --- | --- | --- | --- |
| CET Total (F) | 70.79 | 1 | 3103 | <.001* |  |  |  |
| AN |  |  |  |  | 1920 | 14.87 | 3.31 |
| Others |  |  |  |  | 1185 | 13.85 | 3.27 |
| EDE-Q (W) | 0.36 | 1 | 2586.77 | .551 |  |  |  |
| AN |  |  |  |  | 1920 | 4.05 | 0.79 |
| Others |  |  |  |  | 1185 | 4.03 | 0.76 |
| OCI-R (W) | 54.10 | 1 | 2792.41 | <.001* |  |  |  |
| AN |  |  |  |  | 1920 | 22.85 | 14.65 |
| Others |  |  |  |  | 1185 | 19.21 | 12.56 |
| GAD-7 (F) | 78.15 | 1 | 3103 | <.001* |  |  |  |
| AN |  |  |  |  | 1920 | 10.81 | 5.71 |
| Others |  |  |  |  | 1185 | 8.96 | 5.63 |
| MPS (W) | 81.74 | 1 | 2399.43 | <.001* |  |  |  |
| AN |  |  |  |  | 1920 | 42.52 | 9.18 |
| Others |  |  |  |  | 1185 | 39.35 | 9.71 |
| PHQ-9 (W) | 37.55 | 1 | 2611.86 | <.001* |  |  |  |
| AN |  |  |  |  | 1920 | 14.68 | 6.53 |
| Others |  |  |  |  | 1185 | 13.25 | 6.18 |
| EDQoL (W) | 40.18 | 1 | 2675.88 | <.001* |  |  |  |
| AN |  |  |  |  | 1920 | 1.74 | 0.66 |
| Others |  |  |  |  | 1185 | 1.60 | 0.61 |

Note: *Significant after Bonferroni-Holm Correction. F = Fisher's; W = Welch's; CET = Compulsive Exercise Test; AN = Anorexia Nervosa, EDE-Q = Eating Disorder Examination Questionnaire; OCI-R = Obsessive Compulsive Inventory Revised; GAD-7 = 7 item Generalized Anxiety Disorder scale; MPS = Multidimensional Perfectionism Scale; PHQ-9 = 9 item Patient Health Questionnaire; EDQoL = ED Quality of Life.

Table S3. *First Step of Analyses. Three Linear Regression Analyses with CET as Dependent Variable and BMI, Age, and Sex as Independent Variables*

|  | *b* | SE | $\beta$ | *t* | *p* | *R* | *R*^2^ | Adj. *R*^2^ |
| --- | --- | --- | --- | --- | --- | --- | --- | --- |
| CET |  |  |  |  |  | .21 | .04 | .04 |
| -BMI | -0.10 | .01 | -.21 | -11.71 | <.001* |  |  |  |
| CET |  |  |  |  |  | .05 | .00 | .00 |
| -Age | -0.02 | .01 | -.05 | -3.00 | .003 |  |  |  |
| CET |  |  |  |  |  | .00 | .00 | -.00 |
| -Sex | 0.07 | .41 | .00 | 0.18 | .860 |  |  |  |

Note: *Significant after Bonferroni-Holm correction. CET = Compulsive Exercise Test; BMI = Body Mass Index.

Table S4. S*econd Step of Analyses. Eight Multiple Regression Analyses with CET as Dependent Variable and Psychological Factors together with Covariates as Independent Variables*

|  | *b* | SE | $\beta$ | *t* | *p* | *R* | *R*^2^ | Adj. *R*^2^ | Δ*R^2^* |
| --- | --- | --- | --- | --- | --- | --- | --- | --- | --- |
| CET |  |  |  |  |  | .41 | .17 | .17 | .124 |
| EDE-Q | 1.50 | .07 | .35 | 21.46 | <.001* |  |  |  |  |
| BMI | -0.11 | .01 | -.21 | -12.22 | <.001* |  |  |  |  |
| Age | -0.00 | .00 | -.01 | -0.46 | .648 |  |  |  |  |
| Sex | 0.61 | .37 | .03 | 1.65 | .010 |  |  |  |  |
| CET |  |  |  |  |  | .31 | .10 | .10 | .054 |
| OCI-R | 0.06 | .00 | .24 | 13.55 | <.001* |  |  |  |  |
| BMI | -0.11 | .01 | -.21 | -11.64 | <.001* |  |  |  |  |
| Age | 0.02 | .01 | .06 | 3.06 | .002 |  |  |  |  |
| Sex | 0.24 | .39 | .01 | 0.62 | .539 |  |  |  |  |
| CET |  |  |  |  |  | .29 | .08 | .08 | .039 |
| GAD-7 | 0.12 | .01 | .20 | 11.51 | <.001* |  |  |  |  |
| BMI | -0.10 | .01 | -.20 | -11.06 | <.001* |  |  |  |  |
| Age | 0.01 | .01 | .04 | 2.42 | .016 |  |  |  |  |
| Sex | 0.54 | .39 | .02 | 1.37 | .170 |  |  |  |  |
| CET |  |  |  |  |  | .36 | .13 | .13 | .088 |
| MPS | 0.11 | .01 | .31 | 17.73 | <.001* |  |  |  |  |
| BMI | -0.10 | .01 | -.19 | -10.82 | <.001* |  |  |  |  |
| Age | 0.02 | .01 | .07 | 4.05 | <.001* |  |  |  |  |
| Sex | 0.38 | .38 | .02 | 1.00 | .320 |  |  |  |  |
| CET |  |  |  |  |  | .27 | .07 | .07 | .027 |
| PHQ-9 | 0.09 | .01 | .17 | 9.54 | <.001* |  |  |  |  |
| BMI | -0.11 | .01 | -.21 | -11.75 | <.001* |  |  |  |  |
| Age | 0.01 | .01 | .02 | 1.02 | .309 |  |  |  |  |
| Sex | 0.55 | .39 | .02 | 1.40 | .161 |  |  |  |  |
| CET |  |  |  |  |  | .31 | .10 | .09 | .053 |
| EDQoL | 1.19 | .09 | .23 | 13.46 | <.001* |  |  |  |  |
| BMI | -0.11 | .01 | -.21 | -11.69 | <.001* |  |  |  |  |
| Age | 0.01 | .01 | .02 | 0.97 | .332 |  |  |  |  |
| Sex | 0.52 | .39 | .02 | 1.33 | .182 |  |  |  |  |

*Note*. *Significant after Bonferroni-Holm correction. CET = Compulsive Exercise Test; EDE-Q = Eating Disorder Examination Questionnaire; OCI-R = Obsessive Compulsive Inventory Revised; GAD-7 = 7 item Generalized Anxiety Disorder scale; MPS = Multidimensional Perfectionism Scale; PHQ-9 = 9 item Patient Health Questionnaire; EDQoL = ED Quality of Life; BMI = Body Mass Index.

Table S5. *Complementary Analysis of Step 3, Excluding Male Participants. Multiple Regression Analysis with CET Total as Dependent Variable*

|  | *b* | SE | $\beta$ | *t* | *p* | *R* | *R*^2^ | Adj. *R*^2^ |
| --- | --- | --- | --- | --- | --- | --- | --- | --- |
| CET |  |  |  |  |  | .47 | .22 | .22 |
| EDE-Q | 1.27 | .08 | .30 | 14.98 | <.001* |  |  |  |
| OCI-R | 0.02 | .00 | .09 | 4.42 | <.001* |  |  |  |
| GAD-7 | 0.03 | .01 | .06 | 2.55 | .011 |  |  |  |
| MPS | 0.07 | .01 | .20 | 10.47 | <.001* |  |  |  |
| PHQ-9 | -0.06 | .01 | -.12 | -4.89 | <.001* |  |  |  |
| EDQoL | 0.05 | .12 | .01 | 0.46 | .643 |  |  |  |
| BMI | -0.09 | .01 | -.18 | -10.85 | <.001* |  |  |  |
| Age | 0.02 | .01 | .06 | 3.47 | <.001* |  |  |  |

*Note*. *Significant after Bonferroni-Holm correction. CET = Compulsive Exercise Test; EDE-Q = Eating Disorder Examination Questionnaire; OCI-R = Obsessive Compulsive Inventory Revised; GAD-7 = 7 item Generalized Anxiety Disorder scale; MPS = Multidimensional Perfectionism Scale; PHQ-9 = 9 item Patient Health Questionnaire; EDQoL = ED Quality of Life; BMI = Body Mass Index.
